# Supplementary material for: Circuit analysis reveals a neural pathway for light avoidance in Drosophila larvae
Source: Nat Commun. 2022 Sep 7;13:5274. doi: 10.1038/s41467-022-33059-5 (PMC9452580; doi:10.1038/s41467-022-33059-5)
Supplement: Supplementary file 1 — Supplementary Information [file 41467_2022_33059_MOESM1_ESM.pdf]

Supplementary Figures:

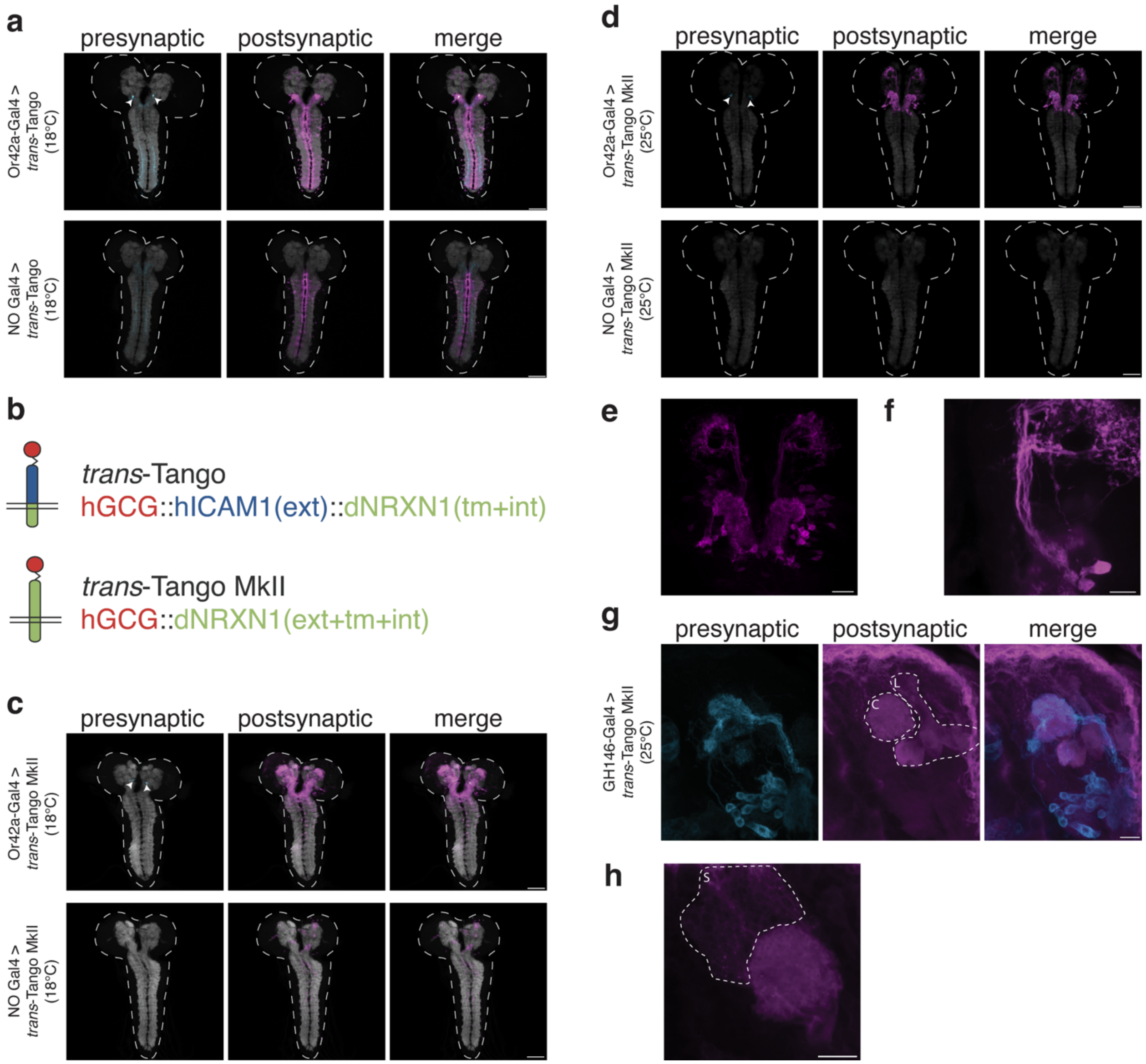

Supplementary Figure 1 ***trans*-Tango MkII eliminates background noise in larval CNS at 25ºC.**

a Postsynaptic partners of Or42a-expressing olfactory receptor neurons are revealed by the original *trans*-Tango. The use of the original *trans*-Tango results in background noise in the

larval VNC in the presence (top) or absence (bottom) of a Gal4 driver. **b** Comparison of the ligand constructs in *trans*-Tango and *trans*-Tango MkII. In *trans*-Tango MkII the dNRXN1 extracellular domain replaces the hICAM1 extracellular domain used in *trans*-Tango. **c, d** *trans*-Tango MkII is used to reveal the postsynaptic partners of Or42a neurons at 18ºC (**c**) and 25ºC (**d**). Background noise in the larval CNS is reduced when *trans*-Tango MkII is used at 18ºC in the presence (top) or absence (bottom) of a Gal4 driver (**c**) and eliminated at 25ºC with (top) or without (bottom) a Gal4 driver (**d**). **e** Close up view of the olfactory circuit in d. **f** Close up view of the processes of olfactory projection neurons in d. Out of six projection neurons, five initially project to the mushroom body calyx, one projects directly to the lateral horn. **g** Subset of the Z-axis sections collapsed to reveal Kenyon cells as postsynaptic partners of GH146-expressing olfactory projection neurons. C denotes the mushroom body calyx, L marks the mushroom body lobes. **h** Subset of the Z-axis sections collapsed to better reveal the somata of Kenyon cells, indicated by S. **a, c, d, e** Presynaptic GFP (cyan), postsynaptic HA (magenta), neuropil (grey). Scale bars, 20µm. Arrowheads indicate the Or42a glomeruli, dashed lines contour the central nervous system. **f-h** Presynaptic GFP (cyan), postsynaptic HA (magenta). Scale bars, 50µm.

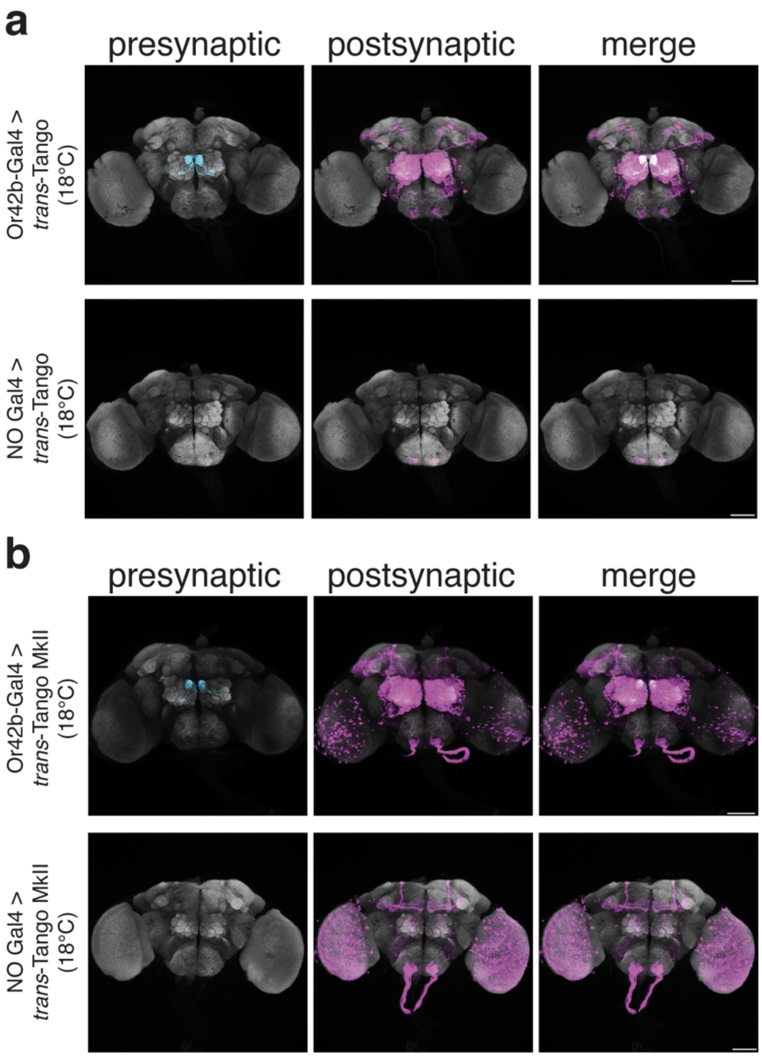

Supplementary Figure 2 ***trans*-Tango MkII exhibits high background noise in adult brains.** **a** Postsynaptic partners of Or42b-expressing olfactory receptor neurons are revealed by *trans*-Tango experiments using the original ligand. The use of the original *trans*-Tango results in little background noise in the adult brain in the presence (top) or absence (bottom) of a Gal4 driver. **b** *trans*-Tango MkII is used to reveal the postsynaptic partners of Or42b neurons. The background noise in the adult brain is high in *trans*-Tango MkII with (top) or without (bottom) a Gal4 driver. Presynaptic GFP (cyan), postsynaptic HA (magenta), neuropil (grey). Scale bars, 50µm. Flies reared at 18ºC.

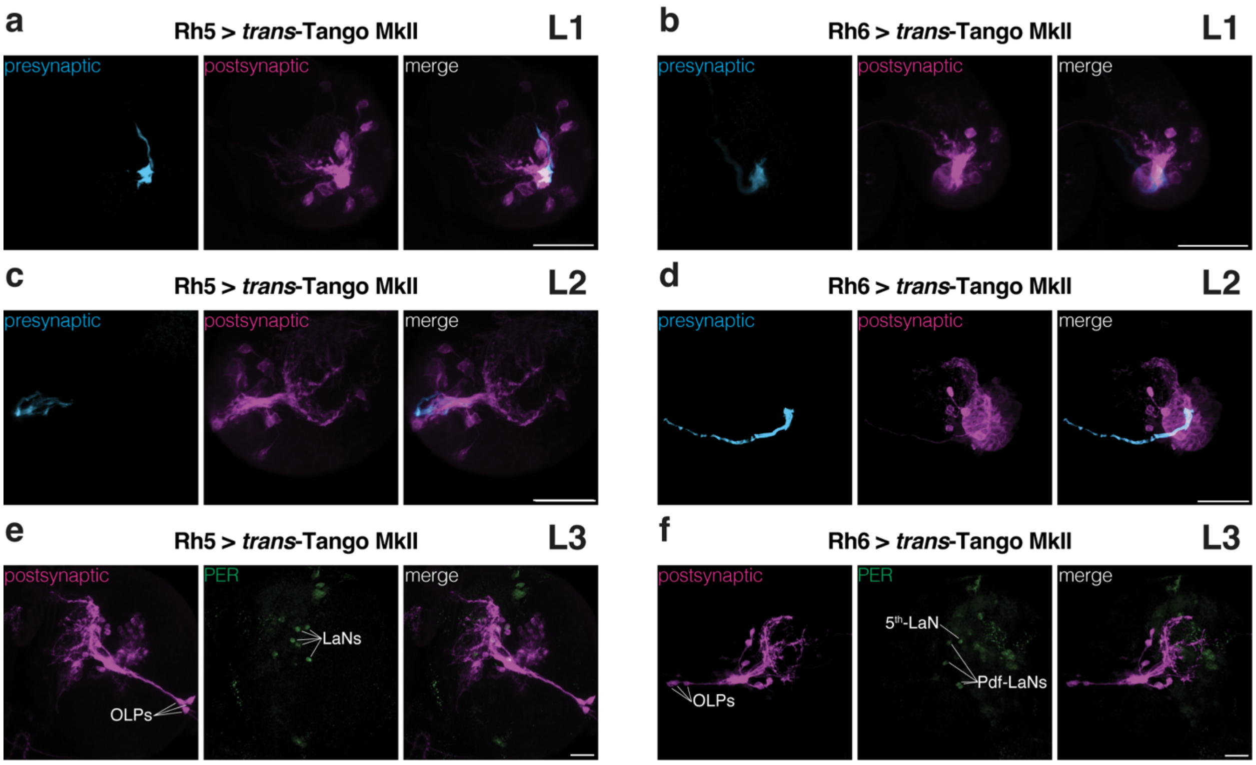

Supplementary Figure 3 **The change in the *trans*-Tango MkII signal throughout development from the first to the third instar larval stage.**

a-f Initiation of *trans*-Tango MkII from Rh5 (**a, c, e**) or Rh6 (**b, d, f**) photoreceptors reveal their postsynaptic partners in first (**a, b**), second (**c, d**) or third (**e, f**) instar larval stages. Note the presence of signal in non-neuronal tissue when Rh6 driver is used in the first (**b**) or second (**d**) larval stages. Remarkably, Pdf-LaNs and the 5th-LaN are revealed as postsynaptic partners of Rh5 photoreceptors (**e**) whereas only Pdf-LaNs are postsynaptic to Rh6 photoreceptors (**f**). Presynaptic GFP (cyan), postsynaptic HA (magenta), PER (green). Scale bars, 20µm. OLP: optic lobe pioneers.

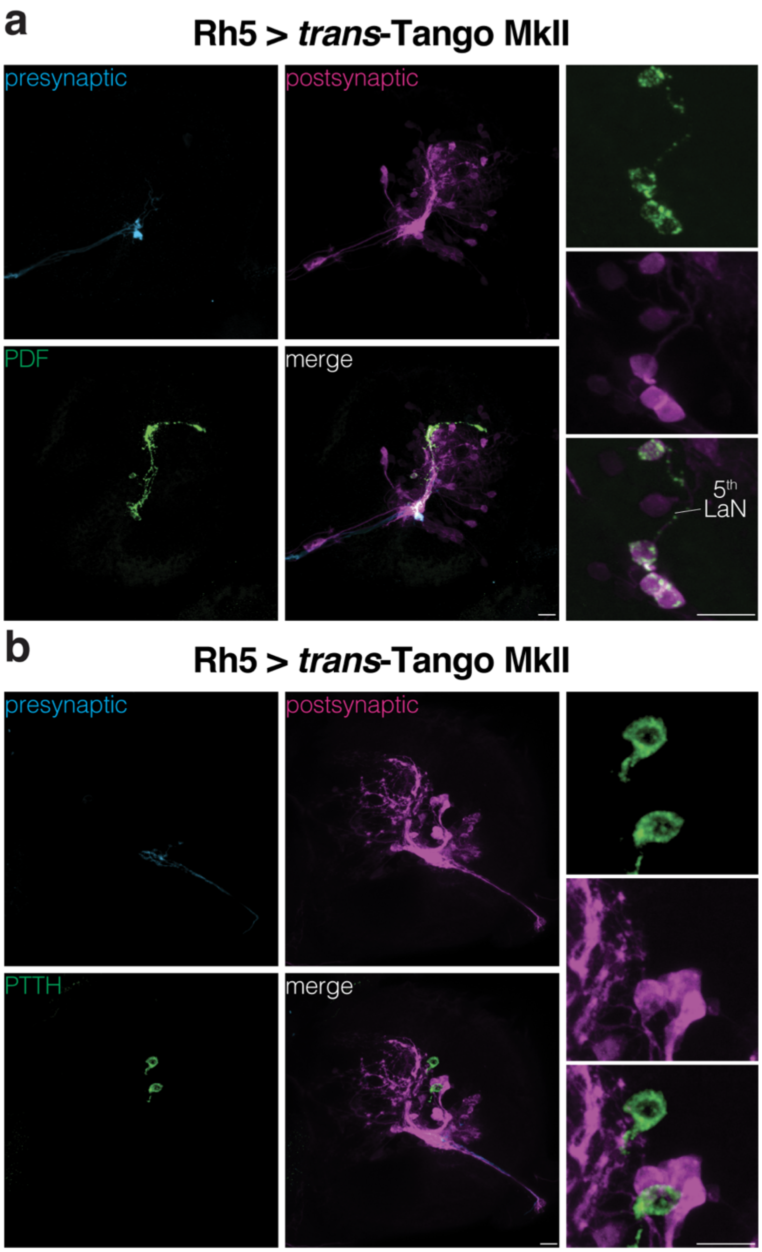

Supplementary Figure 4 **Rh5 photoreceptors are presynaptic to Pdf-LaNs and the 5th-LaN but not to PTTH neurons.**

a Rh5 photoreceptors have direct input onto Pdf-LaNs and the 5th-LaN. **b** PTTH neurons do not receive input from Rh5 photoreceptors. In both panels, presynaptic neurons are revealed by antibody staining against GFP (cyan) and postsynaptic neurons by antibody staining against HA

(magenta). Antibodies against PTTH (**a**) and PDF (**b**) were used to reveal the respective neurons (green). Scale bars, 10μm.

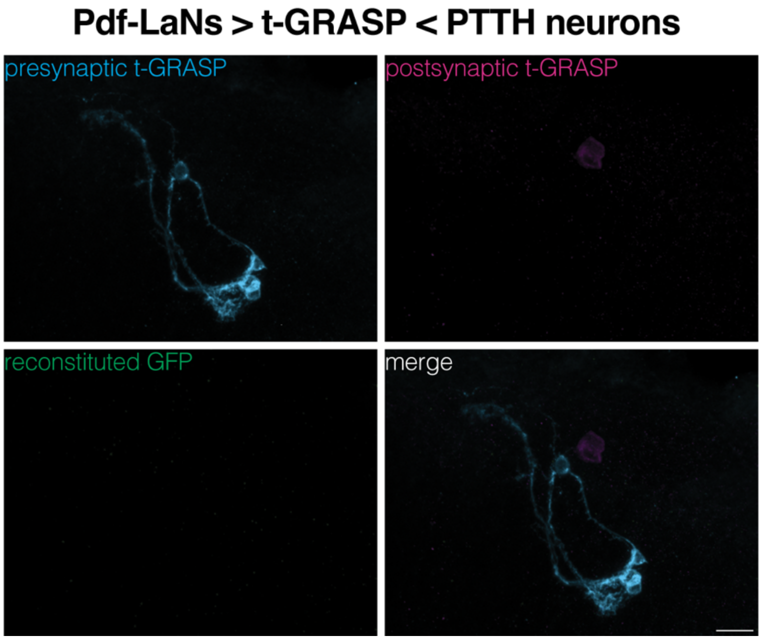

Supplementary Figure 5 **t-GRASP confirms that Pdf-LaNs are not presynaptic to PTTH neurons.**

Presynaptic t-GRASP construct in Pdf-LaNs is revealed by HA staining (cyan). Postsynaptic t-GRASP construct in PTTH neurons is revealed by TLN staining (magenta). No reconstituted GFP signal (green) is observed between Pdf-LaNs and PTTH neurons indicating absence of synaptic connections. Scale bar, 10µm.

a

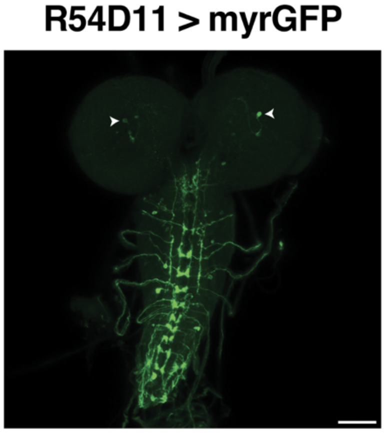

b

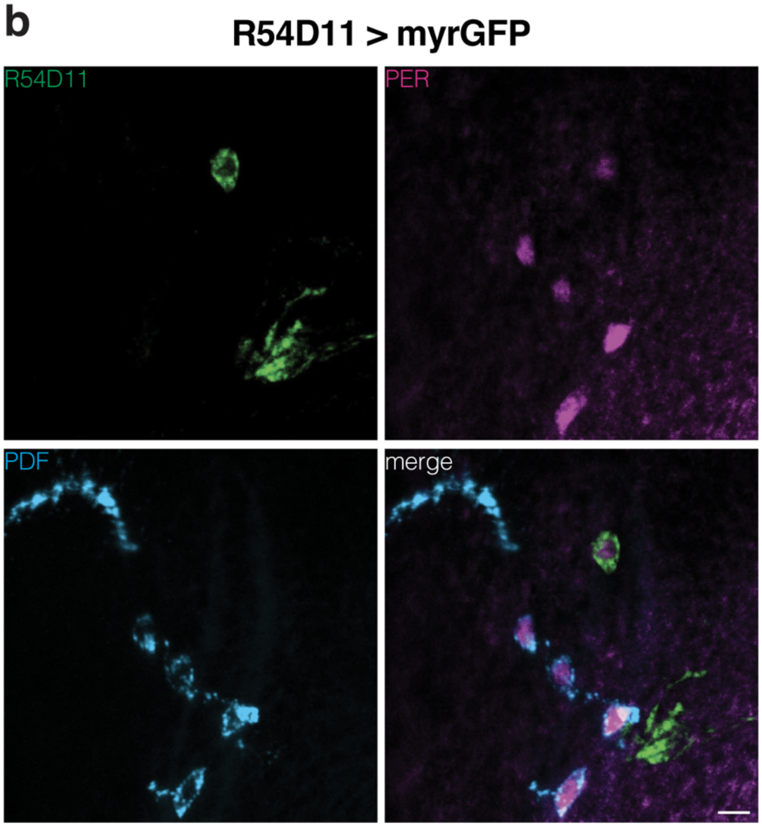

Supplementary Figure 6 **The R54D11-Gal4 driver expresses in the 5th-LaN and in the ventral nerve cord along with weak expression in other brain neurons.**

a R54D11-Gal4 expresses in the brain and the ventral nerve cord. Arrowheads indicate the 5th-LaN. Scale bar 50µm. **b** The expression pattern of R54D11-Gal4 (green) coincides with antibody staining against PER (magenta) but not PDF (cyan), indicating that it labels the 5th-LaN. In both

panels, the expression pattern of R54D11-Gal4 is revealed by antibody staining against GFP (green). Antibodies against PER (magenta) and PDF (cyan) were used to reveal the respective neurons. Scale bar, 5μm.

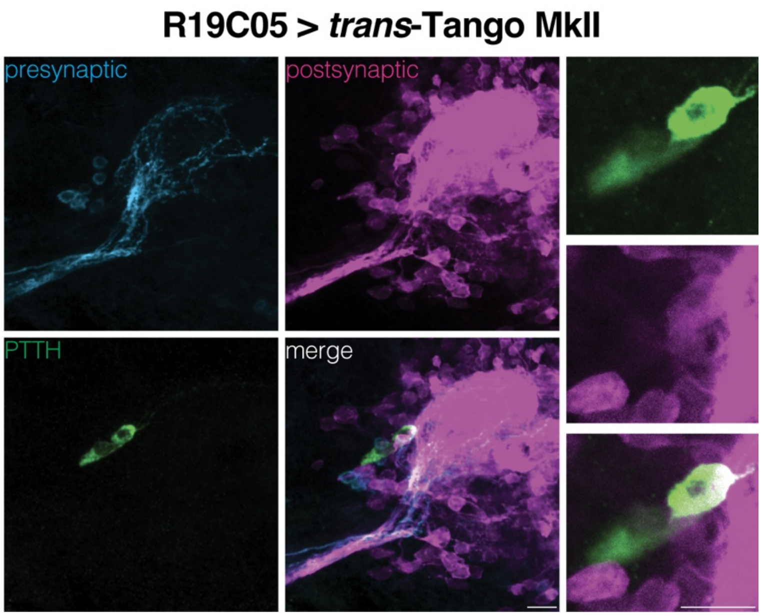

Supplementary Figure 7 **Only one of the PTTH neurons is postsynaptic to the 5th-LaN.** Driving expression of the *trans*-Tango MkII ligand by R19C05-Gal4 confirms that the 5th-LaN connects to one of the PTTH neurons. Presynaptic neurons are revealed by antibody staining against GFP (cyan) and postsynaptic neurons by antibody staining against HA (magenta). Antibody against PTTH was used to reveal the respective neurons (green). Scale bars, 10µm.

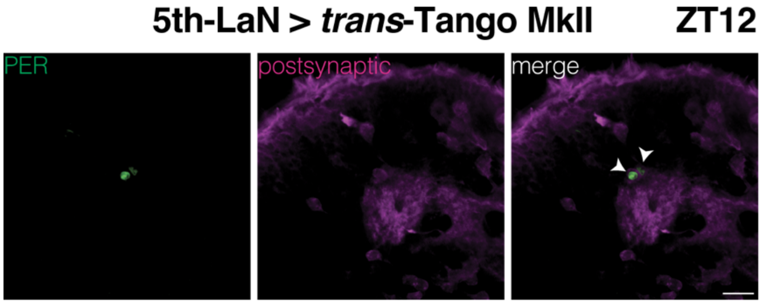

Supplementary Figure 8 **The 5th-LaN is presynaptic to DN2s.**

DN2s receive direct synaptic input from the 5th-LaN (R54D11-Gal4) as revealed by PER staining at ZT12. Postsynaptic neurons are revealed by antibody staining against HA (magenta). Antibody against PER was used to reveal the respective neurons (green). Scale bars, 10µm.

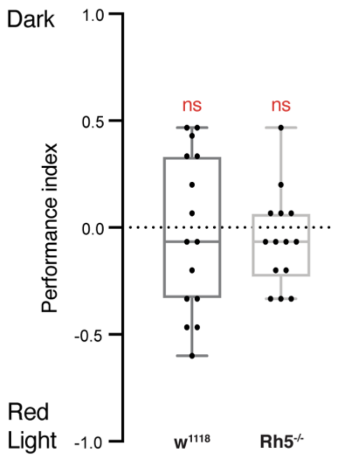

Supplementary Figure 9 **w^{1118} and Rh5$^{-/-}$ mutants do not exhibit any response to red light exposure**

The effect of red light on light avoidance of w^{1118} or Rh5$^{-/-}$ mutants. Boxplots indicate median (middle line), 25th and 75th percentile (box) bars represent maximum and minimum. One sample t-test, ns: not significant. n=15 trials for w^{1118} and n=14 trials for Rh5$^{-/-}$. Source data are provided as a Source Data file.

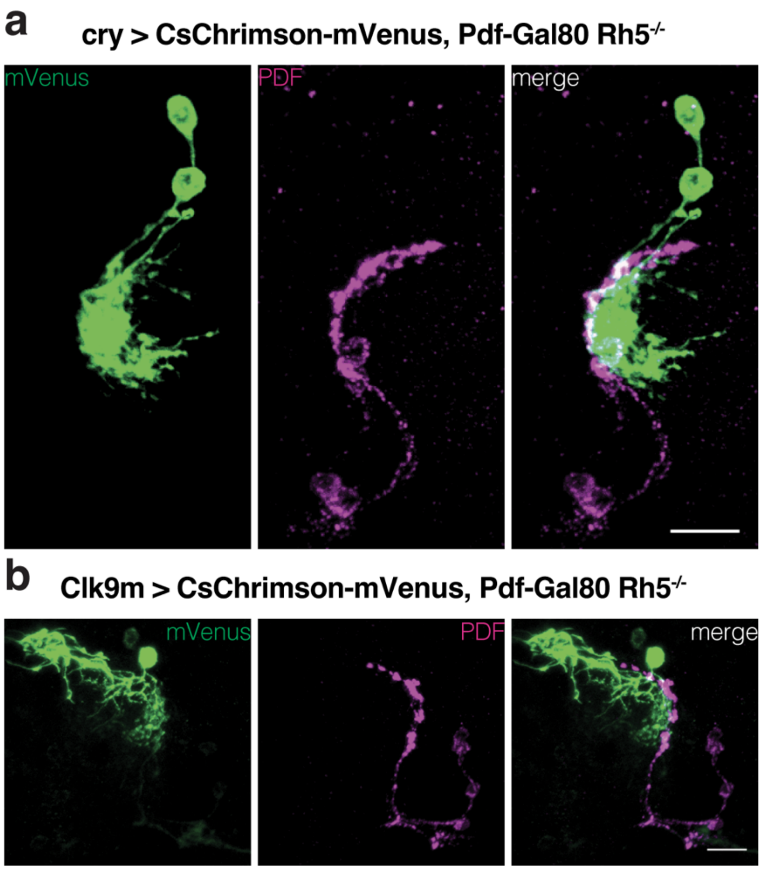

Supplementary Figure 10 **Selective expression of CsChrimson in DN1s and DN2s in Rh5 mutant larvae.**

a Driving of CsChrimson-mVenus by cry-Gal4 in the presence of Pdf-Gal80 in Rh5 mutant larvae results in selective expression in DN1s. **b** Driving of CsChrimson-mVenus by Clk9m-Gal4 in the presence of Pdf-Gal80 in Rh5 mutant larvae results in selective expression in DN2s. Antibody staining against mVenus (green) and PDF (magenta) is shown. Scale bars, 10μm.

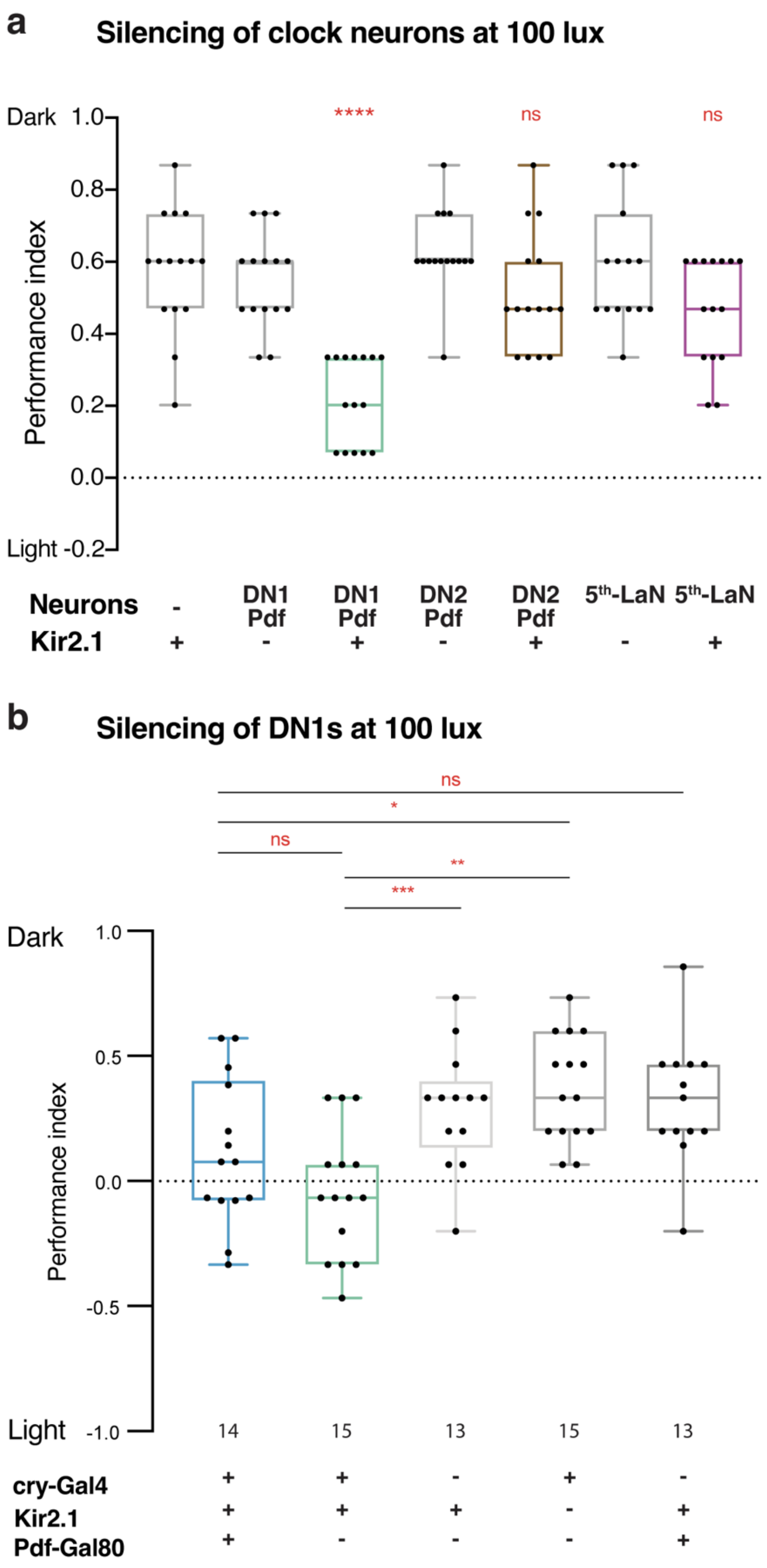

Supplementary Figure 11 **DN1s mediate light avoidance at 100 lux.**

a The effect of Kir2.1-mediated silencing of various clock neuron subsets on light avoidance at 100 lux. Silencing of the DN1s&Pdf-LaNs (cry-Gal4) results in defective light-avoidance, whereas silencing of 5th-LaN or DN2s&Pdf-LaNs (Clk9m-Gal4) has no effect. **b** Selective silencing of DN1s through inclusion of a Pdf-Gal80 together with the cry-Gal4 driver results in significantly reduced light avoidance compared to the driver only control ($p=0.0174$) but not to the no-driver control ($p=0.0674$). Boxplots indicate median (middle line), 25th and 75th percentile (box), bars represent maximum and minimum. One-way ANOVA, ns: not significant, *: $p<0.05$ **: $p<0.01$, ***: $p<0.001$. $n=15$ trials for all genotypes in **a**, the number of trials is indicated under each box in **b**. Source data are provided as a Source Data file.
