# Supplementary material for: Circuit analysis reveals a neural pathway for light avoidance in Drosophila larvae
Source: Nat Commun. 2022 Sep 7;13:5274. doi: 10.1038/s41467-022-33059-5 (PMC9452580; doi:10.1038/s41467-022-33059-5)
Supplement: Supplementary file 3 — Reporting Summary [file 41467_2022_33059_MOESM3_ESM.pdf]

nature portfolio

Corresponding author(s): Gilad Barnea

Last updated by author(s): Jul 29, 2022

Reporting Summary

Nature Portfolio wishes to improve the reproducibility of the work that we publish. This form provides structure for consistency and transparency in reporting. For further information on Nature Portfolio policies, see our Editorial Policies and the Editorial Policy Checklist.

Statistics

For all statistical analyses, confirm that the following items are present in the figure legend, table legend, main text, or Methods section.

n/a	Confirmed	
☐	☒	The exact sample size (*n*) for each experimental group/condition, given as a discrete number and unit of measurement
☐	☒	A statement on whether measurements were taken from distinct samples or whether the same sample was measured repeatedly
☐	☒	The statistical test(s) used AND whether they are one- or two-sided<br>*Only common tests should be described solely by name; describe more complex techniques in the Methods section.*
☒	☐	A description of all covariates tested
☐	☒	A description of any assumptions or corrections, such as tests of normality and adjustment for multiple comparisons
☒	☐	A full description of the statistical parameters including central tendency (e.g. means) or other basic estimates (e.g. regression coefficient) AND variation (e.g. standard deviation) or associated estimates of uncertainty (e.g. confidence intervals)
☐	☒	For null hypothesis testing, the test statistic (e.g. *F*, *t*, *r*) with confidence intervals, effect sizes, degrees of freedom and *P* value noted<br>*Give P values as exact values whenever suitable.*
☒	☐	For Bayesian analysis, information on the choice of priors and Markov chain Monte Carlo settings
☒	☐	For hierarchical and complex designs, identification of the appropriate level for tests and full reporting of outcomes
☒	☐	Estimates of effect sizes (e.g. Cohen's *d*, Pearson's *r*), indicating how they were calculated

Our web collection on statistics for biologists contains articles on many of the points above.

Software and code

Policy information about availability of computer code

Data collection	Zeiss Zen software version 2.1 was used to collect confocal data.
Data analysis	Zeiss Zen software version 2.1 was used for image processing. PRISM 9 from GraphPad was used to analyse photophobia data

For manuscripts utilizing custom algorithms or software that are central to the research but not yet described in published literature, software must be made available to editors and reviewers. We strongly encourage code deposition in a community repository (e.g. GitHub). See the Nature Portfolio guidelines for submitting code & software for further information.

Data

Policy information about availability of data

All manuscripts must include a data availability statement. This statement should provide the following information, where applicable:

- Accession codes, unique identifiers, or web links for publicly available datasets
- A description of any restrictions on data availability
- For clinical datasets or third party data, please ensure that the statement adheres to our policy

All data are available in the main text or the supplementary materials. Source data are provided as a Source Data file.

Field-specific reporting

Life sciences study design

All studies must disclose on these points even when the disclosure is negative.

Sample size	Behavioural assays were run in a similar manner to Mazzoni et al. 2005 and Dettman et al. 2001. Our sample sizes (12-15 animals for each of the 12-15 replicas for each group) exceed both of these articles.
Data exclusions	No data were excluded from the analysis
Replication	All behavioral experiments were run over a three day period with 4-6 replica per day to account for reproducibility concerns. All attempts for replication were successful.
Randomization	Animals from each genotype/treatment were randomly selected from a group of larvae.
Blinding	The experimenter was blind to the genotype of the animals for inhibition experiments. For activation experiments, blinding could not be performed since the experimenter had to avoid chromosomal balancers and the presence of certain balancers indicated which genotype was being used.

Reporting for specific materials, systems and methods

We require information from authors about some types of materials, experimental systems and methods used in many studies. Here, indicate whether each material, system or method listed is relevant to your study. If you are not sure if a list item applies to your research, read the appropriate section before selecting a response.

Materials & experimental systems

n/a	Involved in the study	
☐	☒	Antibodies
☒	☐	Eukaryotic cell lines
☒	☐	Palaeontology and archaeology
☐	☒	Animals and other organisms
☒	☐	Human research participants
☒	☐	Clinical data
☒	☐	Dual use research of concern

Methods

n/a	Involved in the study	
☒	☐	ChIP-seq
☒	☐	Flow cytometry
☒	☐	MRI-based neuroimaging

Antibodies

Antibodies used	The antibodies used in this study are: anti-PDF rabbit, anti-PTTH guinea pig, anti-PER mouse, anti-GFP rabbit (Thermo Fisher Scientific, A11122), anti-HA rat (Roche, 11867423001), anti-Brp mouse (nc82; DSHB), donkey anti-rabbit Alexa Fluor 488 (Thermo Fisher Scientific, A-21206), goat anti-rat Alexa Fluor 555 (Invitrogen, A21434), donkey anti-mouse Alexa Fluor 647 (Thermo Fisher Scientific, A-31571)
Validation	anti-PDF : This antibody was developed and first used by Dircksen, H. et al., Cell and Tissue Research 1987<br>anti-PTTH: This antibody was developed and first used by Yamanaka, N. et al., Science 2013<br>anti-PER: This antibody was developed and first used by Lamaze et al., Curr Biol 2018<br>anti-GFP: This commercial antibody was verified by Relative expression to ensure that the antibody binds to the antigen stated. https://www.thermofisher.com/antibody/product/GFP-Antibody-Polyclonal/A-11122<br>anti-HA The commercially available Anti-HA-Biotin; High Affinity is function tested by Western blot analysis of a HA-tagged fusion protein. https://www.sigmaaldrich.com/US/en/product/roche/roahaha<br>anti-Brp: This antibody was developed by Wagh, et al. 2006 Neuron it is distributed by the DSHB.<br>donkey anti-rabbit Alexa Fluor 488: https://www.thermofisher.com/order/genome-database/generatePdf?productName=Rabbit%20IgG%20(H+L)&assayType=PRANT&productId=A-21206&detailed=true<br>goat anti-rat Alexa Fluor 555: https://tools.thermofisher.com/content/sfs/manuals/mp02794.pdf<br>donkey anti-mouse Alexa Fluor 647: https://www.thermofisher.com/order/genome-database/generatePdf?productName=Mouse%20IgG%20(H+L)&assayType=PRANT&productId=A-31571&detailed=true

Animals and other organisms

Policy information about studies involving animals; ARRIVE guidelines recommended for reporting animal research

Laboratory animals	Drosophila melanogaster, adult males and early third instar stage male and female larvae were used for all experiments. The genotypes of the flies used is included in the methods section of the article.
Wild animals	No wild animal was used

Field-collected samples	No field-collected samples were used.
Ethics oversight	Use of fruit flies in this study did not require ethical approval.

Note that full information on the approval of the study protocol must also be provided in the manuscript.
